# Maintaining trust and seeking support: a qualitative study of family caregivers’ experiences interacting with health care services for home-dwelling older people with mental health problems

**DOI:** 10.1186/s12877-025-05781-4

**Published:** 2025-02-28

**Authors:** Olivia Sissil Sunde, Johanne Alteren, Ole T. Kleiven, Siri Ytrehus

**Affiliations:** 1 Faculty of Health and Social Sciences, Western Norway University of Applied Sciences, Førde, Norway; 2 Faculty of Health Sciences and Social Care, Molde University College Specialized University in Logistics, Molde, Norway

**Keywords:** Family caregivers, Home care, Collaboration, Older people, Mental health problems, Somatic comorbidity, In-depth interview

## Abstract

**Background:**

Enhanced interactions between family caregivers and health care services can improve the care provided to older people and assist caregivers in managing their responsibilities more effectively. However, there are several barriers to involving families in care, and we need more knowledge to understand different aspects of interactions. This study with family caregivers of home-dwelling older people with mental health problems aimed to explore caregivers’ experiences and perceptions of their role in interactions with health care services.

**Methods:**

We conducted individual in-depth interviews with ten family caregivers. Braun and Clark’s thematic approach guided the analysis.

**Results:**

We identified two main themes. The first is the balancing act of managing trust: family caregivers navigating the caring role between empowering autonomy and taking responsibility. The second is the caregiver’s role in a complex care context: family caregivers seeking knowledge, recognition, and support from professional caregivers.

**Conclusions:**

Managing older people’s trust is pivotal to family caregivers’ interactions with health care services. These caregivers carefully balance two crucial aspects: empowering older people’s autonomy and ensuring adequate health care by sharing information. To effectively involve families in the care of older people, this study highlights the need for health care services to consider the competence and time required to balance the dual responsibility of providing optimal care for older people while attending to the needs of family caregivers.

**Supplementary Information:**

The online version contains supplementary material available at 10.1186/s12877-025-05781-4.

## Introduction

Worldwide, welfare states face challenges in meeting the care needs of older populations. This challenge is exacerbated by the ageing demographic, resulting in increased demands for health and care services. Simultaneously, there is a shortage of skilled personnel, intensifying the strain on health care systems. In response, policy-makers have increasingly turned their attention to supporting older populations through a combination of formal and informal care [1, 2]. In Europe, support policies for family caregivers vary. However, the shared goal across countries is to preserve the health and wellbeing of these caregivers while ensuring that they can continue to meet the needs of older populations [3].

This study aims to explore family caregivers’ experiences interacting with health care services regarding care for home-dwelling older people with mental health problems. For clarity, we use “family caregivers” to refer to family and friends providing unpaid care and support. Furthermore, “mental health problems” refer to illnesses such as depression, bipolar disorder, schizophrenia, and anxiety disorders.

## Background

Many older people rely on family caregivers to function daily, and the quality of care provided to home-dwelling older people may depend on family caregiving [4]. While caregiving can provide a sense of purpose and satisfaction to family members [5–7], it is not without challenges. Research has consistently demonstrated the negative impact of caregiving on family members’ health and wellbeing [8–11]. Consequently, supporting family caregivers is essential to help them cope with their responsibilities and maintain their caregiving role [4, 12]. However, a previous literature review reported that family caregivers of older people with mental health problems receive little support when performing their caring role [13].

Despite extensive research on family caregiving, some areas have received less attention. Young’s review [14] highlights that most studies investigating interventions to support family caregivers of older people have focused primarily on those with dementia and cognitive impairment. Other reviews have shown that few studies have explored the experiences and needs of family caregivers of older people with mental health problems [13, 15]. Caregivers of home-dwelling older people may encounter similar challenges and require comparable support, such as information [15]. However, an in-depth understanding of the needs of family caregivers of older people with mental health problems is essential for professionals who collaborate with these caregivers to tailor their support effectively.

Collaboration between families and health care services is presented as a vital solution to meet the needs of older populations. Particularly in mental health care, the literature advocating a recovery-based approach highlights the importance of engaging with families and building on their strengths and resources [16–18]. Studies investigating the perspectives of people experiencing mental health problems describe positive aspects of family involvement in care. Families can play a pivotal role in mental health recovery by providing emotional support and demonstrating affection [19, 20]. Moreover, they provide essential practical support, such as monitoring symptoms, encouraging medication adherence, and facilitating transport to medical appointments [21].

Conversely, family members’ lack of understanding or problematic relationships are described as potential barriers to recovery [19–21]. Furthermore, research indicates that care recipients are afraid of overburdening their family members [21], and receiving help from family members can be experienced as a loss of independence and an invasion of privacy for older people [22].

Although policy and professional guidelines recommend family involvement, the literature suggests that poor implementation is an international problem [23, 24]. Health care professionals report that a lack of prioritization by managers, organizational cultures and paradigms and poor access to training and supervision hamper their involvement in family issues [23, 25]. Moreover, the duty of confidentiality is commonly cited as a challenge in mental health care [24, 26]. These findings suggest a mismatch between what is communicated at the policy level and the actual execution of family involvement in practice.

To improve interactions and establish a good combination of formal and informal care to meet the needs of both older care recipients and their family caregivers, we need to understand different aspects of the collaboration. Our understanding in this area is currently limited, as few studies have explored the experiences and needs of family caregivers of older people with mental health problems in the context of community care. In Norwegian settings, the results of qualitative studies indicate that family caregivers of older people with mental health problems are rarely included as partners in care [27, 28]. Furthermore, the findings from a recent quantitative study show that family members feel a lack of support from health care professionals, and they give a low score on being respected and being invited to take part in care by health care professionals [29].

More research is needed to understand the interactions between family caregivers and professionals to strengthen family members’ ability to cope with caregiving and improve care for older people. Hence, this study provides knowledge of family caregivers’ experiences and perceptions of their caring role in interactions with health care professionals. Every caregiver is at a different point in the experience, with unique views and attitudes towards the situation. Thus, forming an in-depth understanding of their experiences is of notable interest.

## Methods

This study was an exploratory qualitative study conducted within a social constructionist framework. We conducted individual interviews to obtain knowledge of family members’ experiences and views of their caring role when interacting with health care professionals.

### Setting

This study was undertaken in rural municipalities of western Norway. As in other Nordic countries, Norway’s welfare state is characterized by universal and comprehensive public care services, where providing care for older people is a public responsibility [30, 31]. For home-dwelling older people with mental health problems, support is offered through community-based services, which consist of both home health services and mental health care services. While most older people in our study received care from home health services, some were also supported by mental health care services. Our interviews focused primarily on family caregivers’ interactions with community-based services. However, participants shared insight from their interactions with health care professionals in specialized health care services when dealing with complex health conditions in older people. In the presentation of the findings, we specify the nature of these interactions. While nurses provide a large share of home-based services, various professionals are represented, leading us to use the term “health care professionals”.

### Study participants and data collection

The participants were purposefully sampled according to specific criteria: (a) being a caregiver for a home-dwelling older person (aged 65 years and above) with mental health problems and (b) having first-hand experiences interacting with health care services. This study included family caregivers of older people with mental health problems such as depression, bipolar disorder, schizophrenia, and anxiety disorders. We excluded older people with degenerative illnesses, such as Alzheimer’s disease, Huntington’s chorea, and Lewy body dementia.

Participants were recruited with the assistance of managers in community-based services who were acquainted with older people with mental health problems. These managers identified eligible family caregivers and distributed invitation letters to them. Given the challenging nature of the recruitment process, we expanded our reach to include two additional municipalities, resulting in the involvement of five municipalities in the study. Additionally, we extended the recruitment period. Given that managers distributed the initial invitations, the exact number of family caregivers contacted is unknown, but only a few consented to participate. The managers responsible for distributing the invitation letters indicated that some declined due to exhaustion, whereas others did not identify themselves as caregivers of older people with mental health problems. Ultimately, ten family caregivers accepted our invitation to participate in the study.

To gain rich, in-depth knowledge, the interviews were semi-structured with open-ended questions. We developed our interview guide by drawing on insights from previous research on family caregivers’ interactions with health care services. The interview guide was tested in a pilot interview, in line with the recommendations of Kallio et al. [32]. After testing, we made minor changes to the interview guide’s structure and formulated more open-ended questions. The interview guide covered the following topics: family caregivers’ experiences interacting with health care services, their perspectives on essential collaboration situations, and their experiences of support and information. The interviews were conducted by the first author in private, uninterrupted settings, respecting the preference of the participant (participant’s home or workplace, interviewer’s workplace). Data collection spanned from August 2018 to March 2019. Each interview session lasted one to two hours, and the interviews were audiotaped.

### Analysis

We followed Braun and Clark’s [33] latent thematic analysis to identify meanings and patterns across the data. Initially, all interviews, including all spoken words and hesitations, were transcribed by the first author. In the presentation of quotes, we leave out hesitations and words that are unimportant to the meaning. Additionally, ellipses are used to indicate any text that is omitted, whether for clarity or to protect the person not present (the older person receiving care). We read through all the transcribed interviews several times in the first analysis phase and noted immediate thoughts and ideas. The subsequent phase involved inductive coding, where we focused on generating descriptive codes that encapsulated the essence of the participants’ intended meanings. We used NVivo software to code data relevant to our research question, organizing these codes into potential themes and sub-themes. The final phases of our analysis involved an iterative process of defining and naming the emerging themes, writing the research report, and continuously reviewing the themes in connection with the coded data and dataset.

### Reflexivity

Prior understandings and past experiences have likely shaped the interpretation of data and the approach to the study to some extent; hence, reflexivity has been an important principle throughout the research process, inspired by Lincoln and Guba’s recommendations for “trustworthiness” [34]. During the interviews, the first author addressed the credibility criterion by posing open-ended questions, allowing family caregivers to elaborate. Follow-up and clarifying questions were also posed. Furthermore, we met the transferability criterion by presenting comprehensive data descriptions and incorporating participant quotations. To ensure confirmability, all four authors engaged in repeated readings of the transcripts and actively participated in every stage of the analysis, ensuring a nuanced and thorough analysis. In reporting the study, consolidated criteria for reporting qualitative research [35] served as guidance to promote transparency (see File 1).

### Ethics

Ethical approval was obtained from the Norwegian Centre for Research Data (approval number 58265). Each participant provided written consent after receiving information about the study and had the option to withdraw at any time. To safeguard the participants’ confidentiality, the data were stored in double password protected files. Furthermore, we have anonymized data in reporting the findings to protect the personal details of the participants and the older person (care recipient) not present.

## Results

Ten family caregivers aged 40 to 65 years participated in this study. They had all cared for a home-dwelling older person (aged 65–85) with mental health problems for many years (see Table 1 for participant characteristics). Caregiving included providing social and emotional support, monitoring symptoms, organizing professional care, and providing practical help in the home. Furthermore, they provided transport to medical appointments and managed finances, and some occasionally helped with personal care tasks. The older care recipient had defined these participants as primary caregivers with a corresponding right to interact with health care professionals approximately 3 to 15 years ago.

**Table 1:** Participant characteristics

Number of family caregivers by given characteristic	Participants = 10
**Relationship to the older person**	
Son	5
Daughter	3
Nephew	1
Niece	1
**Age**	
40–50	3
51–60	3
61–65	4
**Caring for an older adult experiencing**	
Mental health problems	5
Mental health problems and somatic illness	5
**Duration of care provision**	
**<** 10 years	2
10–20 years	4
20–30 years	2
> 30 years	2

Our data analysis reflects family caregivers’ experiences of conflicting values within their caregiving responsibilities, leading to careful consideration when interacting with health care professionals. The analysis revealed two main themes. The first is the balancing act of managing trust: family caregivers navigating the caring role between empowering autonomy and taking responsibility. The second is the caregiver’s role in a complex care context: family caregivers seeking knowledge, recognition, and support from professional caregivers.

### The balancing act of managing trust: family caregivers navigating the caring role between empowering autonomy and taking responsibility

Family caregivers described their caring responsibilities in interacting with health care professionals as a balancing act between safeguarding the older person’s autonomy and ensuring that he or she received adequate health care. Hence, caregivers constantly adjusted their role following the fluctuating course of the illness, the older person’s preferred level of participation, and their assessment of the older person’s needs.

Family caregivers described an unstable everyday life for themselves and the older person due to unpredictable fluctuations in the older person’s mental health symptoms and somatic comorbidities. The family caregivers emphasized that their close interaction with the older person during long-term health problems had given them valuable insights into how the illness affected the older person’s daily life. Over time, they had become attentive and perceptive in recognizing verbal and non-verbal expressions of emotions indicating mental health deterioration. The quote below illustrates how living in a constantly changing and uncertain situation for many years made family caregivers sensitive to behavioural changes:

When things are unstable, you learn to notice the emotional state. For example, when I came home from school as a child, 2.5 s after the first greeting, I could tell that this was a fine day. You learn to interpret and feel the mood (Participant 10).

The family relationship and close interactions during long-term health problems contributed to family caregivers experiencing a strong emotional attachment between themselves and the older person. In the opinion of family caregivers, these solid emotional bonds created a sense of safety for the older person, allowing the older person to confide in them, share their problems, and rely on them to be responsive to their needs, including making care arrangements. A participant expressed her experience as a trusted caregiver: “She has held on to me, I am her rock, I am the one who sorts things out” (Participant 2).

Family caregivers described how they empowered the older person when engaging with health care services to respect the older person’s self-determination. They looked after the older person’s interests by supporting and strengthening the person in expressing his or her needs or speaking on his or her behalf. A family caregiver gave an example of advocacy for the older person:

I try to look after her interests and do it in the best possible way. After her discharge [from the hospital], health care professionals thought she could not live in her house. That was when the discussion came up […], and I discussed it with her, and she was adamant that she wanted to live in her own house, and I gave a clear message [to the health care professionals] that is how it will be (Participant 7).

Even though family caregivers described the relationship with the older person as open, close, and trusting, there were examples where this was not always the case. Some shed light on how being both a family member and a caregiver could make it difficult for the older person to accept their help. For example, a son recounted how the close family relationship led to his father concealing his mental health problems to protect him and keep him from worrying:

My father is like everyone else who is ill. They must always protect their children. Even though I am an adult, he is still trying to shield me from reality. I have tried to tell him that makes me worry even more (Participant 1).

Other family caregivers shared how the older person’s lack of trust in others, especially during mental health deterioration, created dilemmas in their caring role. For example, family caregivers described interactions where their knowledge of the older person’s situation differed from that of health care professionals. The following quote illustrates family caregivers’ experiences of the older person having challenges communicating his or her mental health needs to health care professionals due to a lack of trust:

If she did not know the home care personnel or those she did not know well enough, then she could pretend that everything was fine, but as soon as they walked out the door, she did not feel so good after all (Participant 8).

On the one hand, family caregivers wanted to share their knowledge with health care professionals because they feared that withholding information could lead to inadequate health care for the older person. On the other hand, sharing too much information with health care professionals could limit the older person’s autonomy in decision-making. Furthermore, if family caregivers shared their unique insight, this could be experienced as a breach of trust by the older adult and harm their relationship. A family caregiver described how protecting the trusting family relationship was a constant consideration when interacting with health care professionals: “I am constantly afraid of breaking the family ties. It is better to be a bit reserved and let him manage by himself” (Participant 9).

Although family caregivers described being reserved, they also shared examples of situations where they felt compelled to take responsibility to ensure that the older person received adequate health care, as illustrated in the quotes below. A daughter shared how she actively assisted her mother in expressing her needs during hospitalization for an acute illness:

My mother was hospitalized, and they had not found out what was wrong with her. We could see she was unwell, so I went over to her and asked, “are you very anxious?”– it was so clear that she had severe anxiety, and they had thought she had a somatic disease. I just said that she has anxiety, and everything fell into place. However, she did not tell them herself (Participant 5).

A son described how he had to take responsibility by involving health care services when his father was at risk of harming himself and was unable to seek help himself:

He has been quite depressed– so in my opinion, he is pretty bad all the time, but to be so depressed that you think about taking your life, you are even further down. There have been episodes where he just wanted to die, and we had to call the doctor to get help assessing whether there was a danger to his life. He had to be forcibly admitted to the hospital (Participant 6).

### The caregiver’s role in a complex care context: family caregivers seeking knowledge recognition and support from professional caregivers

Family caregivers strove to find the best way to provide care in a complex care context, including managing mental health symptoms and somatic illness, respecting the older person’s self-determination, and, at the same time, taking care of themselves and handling other responsibilities.

Family caregivers highlighted the importance of receiving professional support in specific care situations to manage their caregiving responsibilities effectively. Professional knowledge helped them better understand the older person’s experiences with illness, and guidance on how to care for the person in specific situations improved their caregiving skills. In the following quote, a family caregiver describes his experiences of support in being listened to and receiving professional guidance, which allowed him to align his caregiving more closely with the older person’s needs while also considering his capabilities:

There have been many rather harsh conflicts, where I have felt all alone. But then she [the older person] got more contact with the psychiatric nurse and talked to him. I explained [what was going on] and asked if we could do this and that, and we agreed. She could not control our lives. She had to be told that we could not stay home all summer. […] Nevertheless, it was better when I had more contact with the psychiatric nurse, who listened and confirmed what I had done (Participant 3).

Although family caregivers provided examples of health care professionals’ support, they mainly described a lack of recognition and support. Without meaningful engagement from health care professionals, family caregivers experienced a sense of isolation and were compelled to navigate the complexity of caregiving independently. In these situations, their interaction with health care professionals could lead to increased stress in the caregiving role.

Family caregivers’ feelings of isolation were related to experiencing their perspective not being valued or considered when interacting with health care professionals. Communication with health care professionals became an emotionally burdensome caregiving task, leading to distress for family caregivers. Being shut out from sharing their experiences contrasted with their view of themselves as a valuable asset. In particular, family caregivers viewed their unique insights into the older person’s long-term and complex health conditions as valuable for health care professionals tailoring care to the specific needs of the person. One family caregiver provided an example of how sharing her concerns with health care professionals had been significant in ensuring that her mother received adequate health care. However, when health care professionals failed to acknowledge and pay attention to her unique perspective, sharing her valuable insights added another layer of stress to an already demanding caregiving responsibility:

I have an education in health, and I believe I have saved my mother many times because I have observed many mistakes, both with medications and other things. Observations concerning my mother being seriously ill, and the home care nurse thinks it is stomach flu. So, it was hard for me to call them and say I do not think she has the stomach flu. It was unpleasant, but as it turned out, I was right. After all, I am the one who knows my mother best (Participant 4).

Furthermore, family caregivers’ sense of isolation involved feeling left alone with a great care responsibility without health care professionals’ guidance or acknowledgement of their care challenges. A family caregiver described how she was excluded and felt disconnected from interactions with health care professionals, which made the caring role a demanding experience:

First, he cancelled the treatment plan; then, he cancelled that I was his primary caregiver because he was afraid of what that meant, that I could demand, ruin his life, make decisions for him. So then, in a way, the collaboration becomes difficult for the mental health professionals, but it is also difficult for me (Participant 9).

Another family caregiver described a similar experience in which his need for information was overlooked and unaddressed by health care professionals. He lived next door to his mother and cared for her daily. He described being left alone to handle the complexity of caregiving when health care professionals left after a short visit. In his experience, the duty of confidentiality prevented health care professionals from engaging in meaningful interaction:

We can talk, but it is more superficial, like “she is not well”, “now she is more bedridden”. Nothing more. They do not want to talk about it anymore; they stick to their duty of confidentiality, and that is fine. But sometimes I feel that it would be good to know a little more (Participant 8).

Shutting out family caregivers from information also meant that they lacked reassurance about how health care services tailored care to meet the specific needs of the older person. This aspect was emphasized as essential for the wellbeing of family caregivers, as tailored support for the older person could relieve them of their caring responsibilities and alleviate their concerns about their loved one.

## Discussion

This study reveals that family caregivers describe their interactions with health care professionals as a balancing act of managing trust while navigating between safeguarding the older person’s autonomy and ensuring adequate health care. Engaging with health care professionals could help family caregivers cope with their caring responsibilities, but it could also add stress to their caregiving experiences.

We highlight our findings that for family caregivers, managing trust is essential to their interactions with health care services. Theoretical perspectives on interpersonal trust can provide a lens through which to understand family caregivers’ emphasis on managing trust. According to Simpson [36], trust is essential in interpersonal relationships to develop and maintain well-functioning, high-quality relationships. There are various definitions of trust. Most view trust as a psychological state of an individual (the truster) towards a particular partner (the trustee), where the truster needs cooperation to achieve a desired result and accepts vulnerability on the basis of positive expectations of the trustee’s intentions and behaviour [36, 37]. Our results indicate that family caregivers viewed their appointed role as primary caregivers, with the corresponding right to interact with health care professionals, as an act of trust by the older person. Consequently, managing trust became essential for their interactions with health care services.

Our findings suggest that some family caregivers experienced trust as a stable phenomenon, whereas others elaborated on the fragile aspects of trust. Previous studies have shown that different variables, such as situational and relational factors and interindividual differences, interact to shape experiences of trust [38]. Our findings indicate that the level of trust changed with fluctuations in the older person’s mental health problems, which may be related to the truster’s interindividual differences influencing the experience of trust.

This study provides an additional perspective to existing research demonstrating family caregivers’ essential role in providing information [27, 39–41] by shedding light on the dilemma of sharing information and how doing so could challenge trust. Family caregivers perceived sharing information as essential to ensure adequate health care for older adults. However, sharing information with health care services was not straightforward. The experience of family caregivers, as trustees, was that sharing information could be seen as a breach of trust by the older person, posing a risk to their relationship. One possible consequence might be that family caregivers would no longer find themselves in a position to provide help and support.

Knowledge of the fragile aspects of trust and how it can influence family caregivers’ experiences, caring roles, and relationships with older people potentially adds a helpful perspective for health care professionals facilitating family care. Our study shows that promoting family caregiving may be crucial in the complex care context, where older people may have both mental and somatic health problems. For example, family caregivers monitored the older person’s health and provided essential information to health care services when the older person had difficulties expressing his or her needs to health care professionals. This finding suggests that older people with mental health problems have trouble expressing their needs and preferences regarding services, which is consistent with previous research [13].

Health care professionals can provide valuable information and guidance to family caregivers, helping them understand the illness and the older person’s experience. We expect that this understanding can lead to tailored care, better communication, and a more trusting relationship between family caregivers and older people. Notably, the family caregivers who participated in this study had extensive experience with caregiving, which could affect their information needs. For example, many years of caregiving may provide them with a more comprehensive understanding of the health care system compared to those who have recently assumed caregiving responsibilities. As a result, the information needs of family caregivers regarding the health care system may differ significantly. Professional support can help family members cope with their caring responsibilities [40, 42, 43]. Unfortunately, the findings of this study and previous research [13, 28, 29] indicate that many family caregivers have to navigate the complexity of caregiving independently, with little support from professionals.

Trust is an essential element emphasized by health care professionals when building a therapeutic alliance [26, 44] or caring relationship [45] with care recipients. However, this emphasis on establishing and maintaining care recipients’ trust could sometimes hinder health care professionals’ interaction with family caregivers [26, 44]. Consequently, this can result in health care professionals either refraining from providing family caregivers with general information that is not restricted by confidentiality or not receiving information from them. Moreover, there are other barriers to family involvement, such as the duty of confidentiality [24, 26], a lack of prioritization by managers [23], priorities that respond to the care recipient’s immediate health issues over support for family caregivers [44, 46], and lack of knowledge [25, 47].

We recognize that maintaining the trust of older people with mental health problems is a shared priority among family members and health care professionals as they work together to provide care. Consequently, acknowledging trust as an essential aspect of the interaction may be valuable for health care professionals as they balance their responsibility to provide optimal care for older people while attending to family caregivers’ needs.

### Strengths and limitations

One limitation of our study is that recruitment took place with the assistance of managers in community-based services, which may have led to participants who were well engaged with the service. This study may not reflect the views of people who are less engaged in caring for older family members or with services. Moreover, this study was conducted in Norway, which is distinguished by an extensive public care service, where providing care for older people is primarily a public responsibility. This organization of health care services for older people may influence the expectations of family caregivers regarding their collaboration with professionals and could shape the results of this study. Our finding highlighting the phenomenon of a balancing act to manage trust may not necessarily apply to all family caregivers, as different health care systems and different levels of engagement in caring might influence families to focus on other phenomena.

Notably, spouses/partners were also invited to participate in the study, but none accepted. Although the study involved relatively few participants, the interviews provided a rich data source. In our findings, we have presented detailed and thick descriptions, making it possible to transfer information from this study to other settings.

### Implications

To strengthen family members’ ability to cope with care responsibilities and improve care for older people, we recommend educational programmes and that managers of health care services focus on enabling health care professionals to involve families effectively. We propose strengthening their competence around professional, ethical, and legal considerations in involving family members in care. Furthermore, we recommend training in dealing with contradictory principles and values to resolve dilemmas stemming from the dual responsibility of caring for older people and supporting family caregivers while maintaining trust within relationships.

The fluctuations in older people’s mental health problems and the complexity of their care needs influence family caregivers’ need for support, particularly in terms of information and guidance. Hence, the organization of services should focus on flexibility and prioritize the allocation of time to work with families.

This study provides a basis for future studies to explore family caregivers’ experiences interacting with health care services. In future studies, researchers should explore the experiences of spouses/partners, other family members, and friends. The varied dynamics, expectations, and levels of intimacy inherent in different types of relationships could influence their experiences. Additionally, we recommend further research that explores the experiences of home-dwelling older people with mental health problems and their views on family involvement.

## Conclusions

Family caregivers’ interactions with health care professionals involve managing trust while carefully balancing two crucial aspects: empowering older people’s autonomy and ensuring adequate health care by sharing information. Support from health care professionals can help family caregivers adjust their caring role and manage trust. However, caregivers’ interactions with health care professionals can also add stress to their already demanding caregiving experience. Maintaining older people’s trust shapes interactions between family caregivers and health care professionals and requires attention when adjusting the collaboration to ensure both care for older people and support for family caregivers.

## Electronic supplementary material

Below is the link to the electronic supplementary material.

Supplementary Material 1

Supplementary Material 2

## Data Availability

The dataset generated and analysed during the current study is not publicly available because individuals’ privacy could be compromised, but it is available from the corresponding author upon reasonable request.
