# Supplementary material for: Maintaining trust and seeking support: a qualitative study of family caregivers’ experiences interacting with health care services for home-dwelling older people with mental health problems
Source: BMC Geriatr. 2025 Feb 28;25:136. doi: 10.1186/s12877-025-05781-4 (PMC11869469; doi:10.1186/s12877-025-05781-4)
Supplement: Supplementary file 2 — Supplementary Material 2 [file 12877_2025_5781_MOESM2_ESM.docx]

**Interview guide**

| **Topic** | **Interview Questions** | **Follow—up questions** |
| --- | --- | --- |
| Care from the healthcare services | How do you perceive the information you receive from the healthcare services? | - Who gives you information? - Who takes the initiative? - In what situations do you receive information? Have you experienced situations where you wanted information but did not receive it? - Examples of situations where you were satisfied/dissatisfied with the information - What information do you want (content) - Do you have any thoughts on how healthcare professionals can provide information in a better way |
|  | Can you tell me about the help and support you receive for your own sake when interacting with the healthcare services? | - Examples of a situation where you received help/support, description of the situation, and what help you received - How did you receive help, who took the initiative, or how did you go about getting help - What expectations do you have of the healthcare professional role towards you |
| Interaction with healthcare professionals | Can you describe situations where you believe it is vital for the professionals and family members to collaborate? | - Specific tasks or situations - Assessment of the purpose of the collaboration, who it is essential for, and why - Do you have any thoughts on how the healthcare services can be improved through collaboration with family members, suggestions for improvement |
|  | Can you describe your experiences with collaborating with healthcare professionals? Examples of good/less good collaboration? | - Examples of a situation that you experienced as good/less good - is there something you would highlight as particularly important for your experience of the collaboration being good/less good - How do you assess your ability to influence collaboration situations - What do you believe is essential for the collaboration with the healthcare professionals to work well - What promotes and inhibits collaboration |
